# Notochordal cell disappearance and modes of apoptotic cell death in a rat tail static compression-induced disc degeneration model

**DOI:** 10.1186/ar4460

**Published:** 2014-01-29

**Authors:** Takashi Yurube, Hiroaki Hirata, Kenichiro Kakutani, Koichiro Maeno, Toru Takada, Zhongying Zhang, Koji Takayama, Takehiko Matsushita, Ryosuke Kuroda, Masahiro Kurosaka, Kotaro Nishida

**Affiliations:** 1 Department of Orthopaedic Surgery, Kobe University Graduate School of Medicine, 7-5-1 Kusunoki-cho, Chuo-ku, Kobe 650-0017, Japan

## Abstract

**Introduction:**

The intervertebral disc has a complex structure originating developmentally from both the mesenchyme and notochord. Notochordal cells disappear during adolescence, which is also when human discs begin to show degenerative signs. During degeneration later in life, disc cells decline because of apoptosis. Although many animal models have been developed to simulate human disc degeneration, few studies have explored the long-term changes in cell population and phenotype. Our objective was to elucidate the time-dependent notochordal cell disappearance and apoptotic cell death in a rat tail static compression-induced disc degeneration model.

**Methods:**

Twenty-four 12-week-old male Sprague–Dawley rat tails were instrumented with an Ilizarov-type device and loaded statically at 1.3 MPa for up to 56 days. Loaded and distal-unloaded discs were harvested. Changes in cell number and phenotype were assessed with histomorphology and immunofluorescence. Apoptosis involvement was determined with terminal deoxynucleotidyl transferase dUTP nick-end labeling (TUNEL) staining and immunohistochemistry.

**Results:**

The number of disc nucleus pulposus and annulus fibrosus cells decreased with the loading period; particularly, the decrease was notable at day 7 in larger, vacuolated, cytokeratin-8- and galectin-3-co-positive cells, indicating notochordal origin. Subsequently, the proportion of cells positive for TUNEL and cleaved caspase-3, markers of apoptosis induction, increased from day 7 through day 56. Although the percentage of cells immunopositive for cleaved caspase-8, a marker of apoptosis initiation through the death-receptor pathway, increased only at day 7, the percentage of cells immunopositive for cleaved caspase-9 and p53-regulated apoptosis-inducing protein 1 (p53AIP1), markers of apoptosis initiation through the p53-mediated mitochondrial pathway, increased from day 7 through day 56. The percentage of cells immunopositive for B-cell lymphoma 2 (Bcl-2) and silent mating type information regulation 2 homolog 1 (SIRT1), antiapoptotic proteins, decreased consistently with compression.

**Conclusions:**

This rat tail model mimics notochordal cell disappearance and apoptotic cell death in human disc aging and degeneration. Sustained static compression induces transient activation of apoptosis through the death-receptor pathway and persistent activation of apoptosis through the p53-mediated mitochondrial pathway in disc cells. The increased proapoptotic and decreased antiapoptotic proteins observed at all time points signify static compression-induced disc cell death and degeneration.

## Introduction

Back pain is a global health problem. In the United States, a reported 3-month prevalence of back pain was 30.7% [1], and estimated direct and indirect costs were $90.7 billion [2] and $19.8 billion [3], respectively. The cause of back pain is multifactorial; however, intervertebral disc degeneration is associated with back pain, as shown by the observation that UK women with advanced disc degeneration have 3.2 higher odds of manifesting low back pain [4].

The intervertebral disc has a complex structure with the nucleus pulposus (NP) encapsulated by the end plates and the annulus fibrosus (AF). Whereas the AF arises from the mesenchyme [5,6], the NP originates from the notochord [7]. Notochordal cells exist during only approximately the first 10 years of human life, and are then replaced by non-notochordal, chondrocyte-like cells of unknown provenance [5,6].

Intervertebral disc degeneration is characterized by extracellular matrix degradation and decreased cellularity [8]. Morphologic and biochemical evidence suggests that the disc degenerates starting from early childhood [9,10], and these changes are generally more severe in the NP than in the AF [10]. In the NP, obvious clefts and radial tears can occur in ages 11 through 16 years [10]. Aggrecan biosynthesis and type 2 procollagen content are highest in ages 5 years and younger and diminish by 5 to 15 years, and denatured type 2 collagen percentage is lowest in ages 5 years and younger and increases thereafter [9]. Programmed cell death, apoptosis, also increases substantially in ages 11 to 16 years, associated with notochordal cell disappearance and chondrocyte proliferation [10]. These lines of evidence suggest a possible link between the loss of notochordal cells and the pathogenesis of disc degeneration [5].

**Figure 1:**
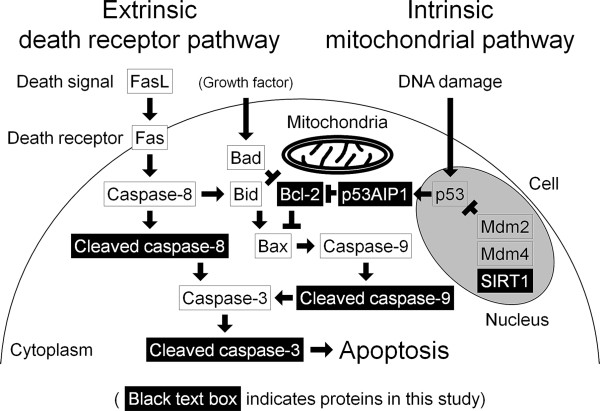
**Schematic illustration summarizing apoptotic signaling through the death receptor pathway and the mitochondrial pathway.** The death receptor pathway is initiated by extrinsic signals such as the binding of death-inducing ligands (for example, Fas ligand (FasL)), to cell-surface receptors (for example, Fas). This complex cleaves the pro-form of initiator caspase-8, followed by direct or indirect (via the mitochondrial signaling loop) cleavage of the pro-form of effector caspase-3. The mitochondrial pathway is initiated by intrinsic signals (for example, DNA damage). DNA damage acetylates p53, which is deacetylated by the silent mating-type information regulation 2 homolog 1 (SIRT1). DNA damage phosphorylates p53 by dissociation of the complex of p53 and its negative regulators, murine double minutes 2 (Mdm2) and 4 (Mdm4). The p53 phosphorylation at serine 46 induces p53-regulated apoptosis-inducing protein 1 (p53AIP1) expression. p53AIP1 interacts with B-cell lymphoma 2 (Bcl-2). Imbalanced Bcl-2 family members (such as proapoptotic Bcl-2-associated X protein (Bax), Bcl-2-associated agonist of cell death (Bad), and BH3-interacting domain death agonist (Bid) and antiapoptotic Bcl-2), induce mitochondrial membrane permeabilization, cytochrome *c* release, and cleavage of the pro-form of initiator caspase-9 followed by caspase-3 cleavage, resulting in apoptosis.

Apoptosis acts as a quality control mechanism for the maintenance of tissue homeostasis by eliminating defective cells [11]. Cells undergo apoptosis through two major pathways: the death receptor pathway and the mitochondrial pathway (Figure 1) [12]. The death receptor pathway is initiated by apoptotic stimuli comprising extrinsic signals such as the binding of death-inducing ligands (for example, Fas ligand (FasL)), to cell surface receptors (for example, Fas). This complex activates initiator caspases, primarily caspase-8, followed by direct or indirect (via the mitochondrial signaling loop) activation of effector caspases, predominantly caspase-3. The mitochondrial pathway is initiated by intrinsic signals (for example, DNA damage), induced by diverse apoptotic stimuli, which converge at the mitochondria. DNA damage acetylates p53, which is deacetylated by the silent mating type information regulation 2 homolog 1 (SIRT1) [13,14]. DNA damage phosphorylates p53 by dissociation of the complex of p53 and its negative regulators, murine double minutes 2 and 4 [15]. On severe DNA damage, serine 46 on p53 is phosphorylated, and p53-dependent apoptosis is induced—only when p53-regulated apoptosis-inducing protein 1 (p53AIP1) is expressed [16]. p53AIP1 is a pivotal mediator of apoptosis through the mitochondrial pathway, interacting with B-cell lymphoma 2 (Bcl-2) [17]. Imbalanced Bcl-2 family members, such as pro apoptotic Bcl-2-associated X protein (Bax), Bcl-2-associated agonist of cell death, and BH3-interacting domain death agonist and antiapoptotic Bcl-2, induce mitochondrial membrane permeabilization, cytochrome *c* release, and initiator caspase-9 activation, followed by effector caspase-3 activation, resulting in apoptosis.

A high incidence of apoptotic cells is observed in human aged and degenerated discs [18]. However, the progression of apoptosis and its practical significance in intervertebral disc degeneration still remain unclear.

Systematic analysis of the disc degeneration mechanism by using human specimens is difficult because of its diverse etiologies, such as mechanical stress, injury, inflammation, smoking, nutrient loss, and aging [8]; therefore, reliable animal models of disc degeneration are required. Rodents retain notochordal cells in the NP throughout their lifetime [6]. Although this limits relevance to the human condition, studies using rodent models have provided considerable insights into the notochordal cell-related pathogenesis of disc degeneration [5].

We previously reported a rat tail model of disc degeneration induced by a common induction method—mechanical loading—which mimics extracellular matrix metabolic imbalances in human disc degeneration [19,20]. The observed imbalances of degradative enzymes and their inhibitors and the net effect on aggrecanolysis under sustained static compression are consistent with human evidence [21,22]. This similarity with the human condition conveys the primary advantage of static compression for longitudinal investigation of disc degeneration.

Static compression decreases disc cell numbers, simulating human degeneration [23-27]. The primary question in this study was why disc cells decline in number under static compression despite limited trauma to the disc [19,20], unlike annular puncture [6]. The mechanism of static compression-induced decreased cellularity has been partially explained by increased apoptosis [23-27] through the mitochondrial pathway [27]. However, the long-term aspects of apoptotic signaling and the balance between proapoptotic and antiapoptotic proteins throughout the degenerative process have not been studied. The role of notochordal cell disappearance in this process has also remained undetermined. Therefore, we undertook an *in vivo* approach by using the rat tail static compression-induced disc degeneration model to elucidate the time-dependent notochordal cell disappearance and apoptotic cell death.

## Materials and methods

All animal procedures were performed under the approval and guidance of the Animal Care and Use Committee at Kobe University Graduate School of Medicine.

### Animals and surgical procedure

**Figure 2:**
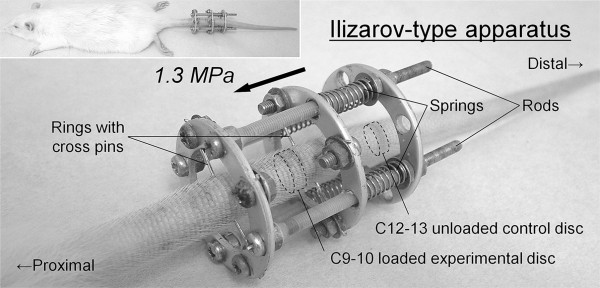
Whole and close-up views of a rat tail instrumented with an Ilizarov-type loading device.

In total, 24 12-week-old male Sprague–Dawley rats (CLEA Japan, Tokyo, Japan), ranging in weight from 452 g to 509 g, were used. Rats are reported to reach approximately 90% of skeletal maturity 12 weeks after birth [28]. Rat tails were affixed with an Ilizarov-type apparatus with springs, a type similar to that of Iatridis and colleagues [29], between the caudal (C) vertebrae 8 and 10, as described in our previous articles (Figure 2) [19,20,30]. In brief, two-cross 0.7-mm diameter Kirschner wires were inserted percutaneously into each vertebral body perpendicular to the tail’s axis and attached to aluminum rings. Rings were connected longitudinally with four threaded rods. Four 0.50-N/mm calibrated springs were installed over each rod. After instrumentation, axial force was applied from the distal side to produce a calculated compressive stress of 1.3 MPa. This stress, corresponding closely to transient disc loading force produced by lifting a moderate weight in the human lumbar spine, is shown to induce morphologic and biochemical disc degeneration [23,24].

After surgery, rats were loaded for 0 (sham), 7, 28, or 56 days and euthanized. Rat tails with the compressive apparatus unloaded for up to 56 days were used as the sham group. In 24 rats (*n* = 6/time point), C9-10, the distal loaded disc, and C12-13, the unloaded internal control disc [19,20], were harvested for histologic assessment.

### Paraffin-embedded disc tissue preparation

Loaded and unloaded vertebral body–disc–vertebral body units were excised, fixed in 4% paraformaldehyde, decalcified in 10% ethylenediaminetetraacetic acid, embedded in paraffin, sectioned from the mid-sagittal plane at 5-μm thickness, and prepared for histologic analysis.

### Hematoxylin and eosin staining

Sections were stained with hematoxylin and eosin according to a standard procedure and photographed by using the BZ-9000 microscope (Keyence, Osaka, Japan).

### Immunofluorescence

Sections were incubated with 1:20-diluted mouse-monoclonal anti-cytokeratin-8 (sc-101459; Santa Cruz Biotechnology, Santa Cruz, CA, USA) and 1:50-diluted rabbit-polyclonal anti-galectin-3 (sc-20157; Santa Cruz Biotechnology) antibodies at 4°C overnight, and subsequently treated with 1:200-diluted Alexa Fluor 488-labeled anti-mouse and 568-labeled anti-rabbit antibodies (Molecular Probes, Eugene, OR, USA) at room temperature for 2 hours. Two μg/ml of 4′,6-diamidino-2-phenylindole (DAPI) (Molecular Probes) was used for nuclear counterstaining. Images were obtained by using the BZ-9000 microscope (Keyence). DAPI-positive nuclei were counted in five random high-power fields (×400) within both NP and AF by using the BZ-9000 analysis software (Keyence). The counts were performed in random duplicate sections. The total cell population was calculated as the mean population of the six rats corresponding to each time point. Cells co-immunopositive for cytokeratin-8 and galectin-3 were similarly counted, and the percentage of these cells in total cells was calculated.

### Terminal deoxynucleotidyl transferase dUTP nick-end labeling staining

Sections were stained by using a fluorescent terminal deoxynucleotidyl transferase dUTP nick-end labeling (TUNEL) assay kit (Roche Diagnostics, Mannheim, Germany) with counterstaining for DAPI. The percentage of TUNEL-positive cells was calculated as described earlier.

### Immunohistochemistry

Sections were incubated with 1:50-diluted rabbit-polyclonal anti-cleaved caspase-3 (9661; Cell Signaling Technology, Danvers, MA, USA), 1:10-diluted rabbit-monoclonal anti-cleaved caspase-8 (8592; Cell Signaling Technology), 1:10-diluted rabbit-polyclonal anti-cleaved caspase-9 (9507; Cell Signaling Technology), 1:50-diluted rabbit-polyclonal anti-p53AIP1 (sc-22761; Santa Cruz Biotechnology), 1:10-diluted mouse-monoclonal anti-Bcl-2 (sc-7382; Santa Cruz Biotechnology), or 1:100-diluted rabbit-polyclonal anti-SIRT1 (LS-B1564; LifeSpan Biosciences, Seattle, WA, USA) antibody at 4°C overnight, and subsequently treated with a peroxidase-labeled anti-mouse or anti-rabbit antibody (Nichirei Bioscience, Tokyo, Japan) at room temperature for 30 minutes. The blot was developed by using peroxidase substrate 3,3′-diaminobenzide. Counterstaining was performed with hematoxylin, except for SIRT1, which was performed with methyl green because of its nuclear localization [13,14]. Parallel sections treated with normal IgG (Dako, Glostrup, Denmark) at equal protein concentrations were used as negative controls. Positive brown staining was calculated as the percentage of immunopositive cells to total cell population measured by counting the nuclei.

### Statistical analysis

Data are expressed as mean ± standard deviation. Two-way analysis of variance with the Tukey-Kramer *post hoc* test was used to investigate changes for effects of compression (loaded and unloaded) and time (0, 7, 28, and 56 days). We analyzed 6 loaded and 6 unloaded discs from 6 rats (1 disc each) for each of the four time points (total 24 loaded and 24 unloaded discs from 24 rats). Statistical significance was assessed with *P* < 0.05 by using PASW Statistics 18 (SPSS, Chicago, IL, USA).

## Results

All animals tolerated surgery well and gained body weight throughout the duration of the experiment (543 to 614 g at day 56). All springs maintained their compressive length and fully recovered immediately after release, indicating sustained axial loading. No signs of infection, skin necrosis, neurologic problems, or instrument failure were observed.

We included normal IgG negative controls and appropriate positive controls in immunofluorescence, immunohistochemistry, and TUNEL staining. As expected, the IgG negative controls showed no staining, and strong staining signals were present in the positive controls.

### Sustained static compression induces a disproportionately large decrease in intervertebral disc cells with a notochordal phenotype

**Figure 3:**
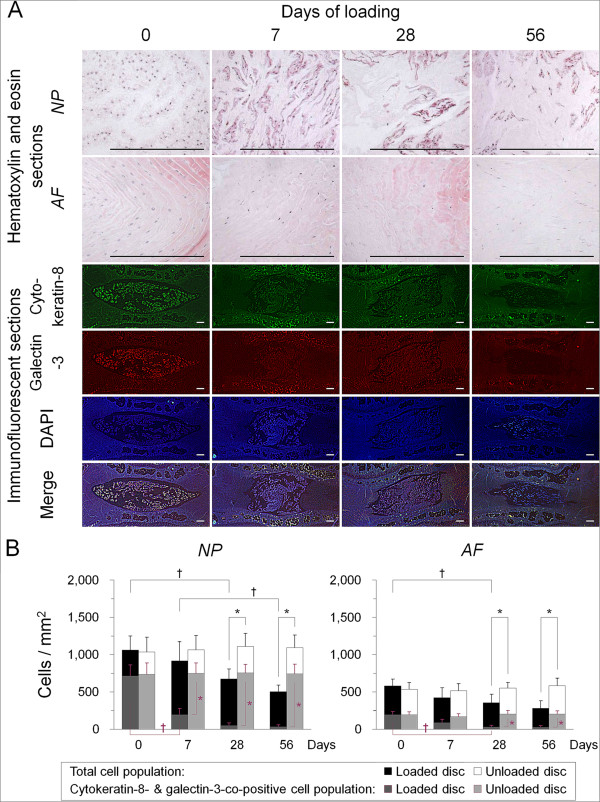
**Sustained static compression induces a disproportionately large decrease in intervertebral disc cells with a notochordal phenotype. (A)** Representative hematoxylin and eosin sections and immunofluorescent sections for cytokeratin-8, galectin-3, 4′,6-diamidino-2-phenylindole (DAPI), and merged signals in the loaded disc nucleus pulposus (NP) and annulus fibrosus (AF) at 0, 7, 28, and 56 days of loading (bars, 200 μm). **(B)** Changes in the population of DAPI-positive total cells and cytokeratin-8- and galectin-3-co-positive cells, identified as notochordal cells, in the NP and AF at 0, 7, 28, and 56 days of loading. Data are mean ± standard deviation (*n* = 6). Two-way analysis of variance with the Tukey-Kramer *post hoc* test was used. *P < 0.05 when compared between loaded and unloaded conditions. ^†^*P* < 0.05 when compared between different time points.

First, to characterize disc cellular composition, we performed hematoxylin and eosin staining. In the NP, larger, vacuolated, notochordal cells were frequently observed at day 0 but largely disappeared from day 7, whereas smaller, round, chondrocyte-like cells clustered but were found throughout the study duration. In the AF, evenly distributed, fibroblast-like cells were observed at day 0 but subsequently decreased, and larger, round, chondrocyte-like cells appeared (Figure 3A). Next, to determine the population and localization of notochordal cells in the disc, we performed immunofluorescence. We selected cytokeratin-8 and galectin-3 as likely markers of notochordal cells from reported evidence [5]. Cytokeratin-8 showed cytoplasmic localization. Galectin-3 demonstrated nuclear as well as cytoplasmic localization. Immunopositivity was slightly higher for galectin-3 than for cytokeratin-8. Immunoreactivity for cytokeratin-8 and galectin-3 was markedly higher in the NP than in the AF (Figure 3A). Cell count analysis revealed that the number of DAPI-positive disc cells decreased with the loading period (*P* < 0.05 at days 28 and 56), up to 47.5% in the NP and 48.5% in the AF at day 56 compared with at day 0 (Figure 3B). Cells co-immunopositive for cytokeratin-8 and galectin-3, identified as notochordal cells, accounted for 67.4% of total NP cells at day 0 but significantly decreased at day 7 and later time points (*P* < 0.05)—21.5% at day 7, 7.7% at day 28, and 6.9% at day 56. Cytokeratin-8- and galectin-3-co-positive cells occupied 34.0% of total AF cells at day 0 but subsequently decreased with significance at days 28 and 56 (*P* < 0.05) (Figure 3B).

### Sustained static compression induces apoptotic cell death in the intervertebral disc

**Figure 4:**
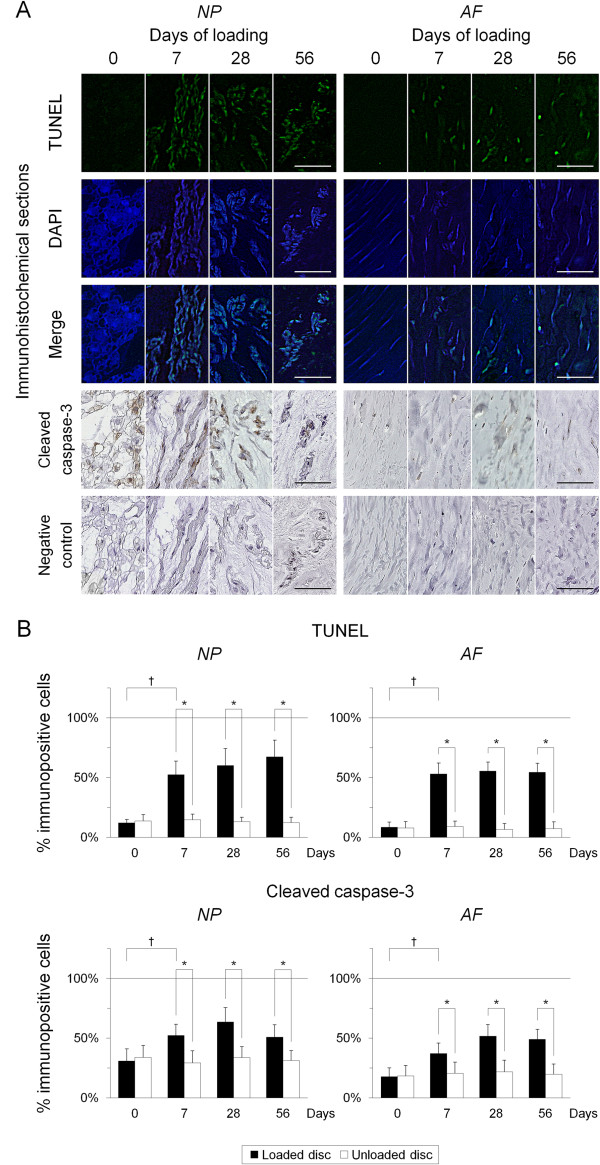
**Sustained static compression induces apoptotic cell death in the intervertebral disc. (A)** Representative sections for terminal deoxynucleotidyl transferase dUTP nick-end labeling (TUNEL), 4′,6-diamidino-2-phenylindole (DAPI), and merged signals and immunohistochemical sections for cleaved caspase-3 and negative control in the loaded disc nucleus pulposus (NP) and annulus fibrosus (AF) at 0, 7, 28, and 56 days of loading (bars, 50 μm). **(B)** Changes in the percentage of TUNEL-positive cells and cleaved caspase-3-positive cells for in the NP and AF at 0, 7, 28, and 56 days of loading. Data are mean ± standard deviation (*n* = 6). Two-way analysis of variance with the Tukey-Kramer *post hoc* test was used. *P < 0.05 when compared between loaded and unloaded conditions. ^†^*P* < 0.05 when compared between different time points.

To clarify the involvement of apoptosis in static compression-induced disc cell loss, we performed TUNEL staining and immunohistochemistry for cleaved caspase-3. TUNEL-reactivity was localized in the nucleus (Figure 4A). Immunoreactivity for cleaved caspase-3 was localized in the cytoplasm and partially in the nucleus, and was stronger in the NP than in the AF (Figure 4A). In cell count analysis, the percentage of TUNEL-positive cells was low at day 0 but significantly increased from day 7 through day 56 in the NP and AF (*P* < 0.05) (Figure 4B). Surprisingly, the percentage of cells immunopositive for cleaved caspase-3 was higher than that for TUNEL at day 0, particularly in the NP. The percentage of cleaved caspase-3-positive cells significantly increased from day 7 through day 56 in the NP and AF (*P* < 0.05) (Figure 4B).

### Sustained static compression induces transient activation of apoptosis through the death receptor pathway and persistent activation of apoptosis through the p53-mediated mitochondrial pathway in intervertebral disc cells

**Figure 5:**
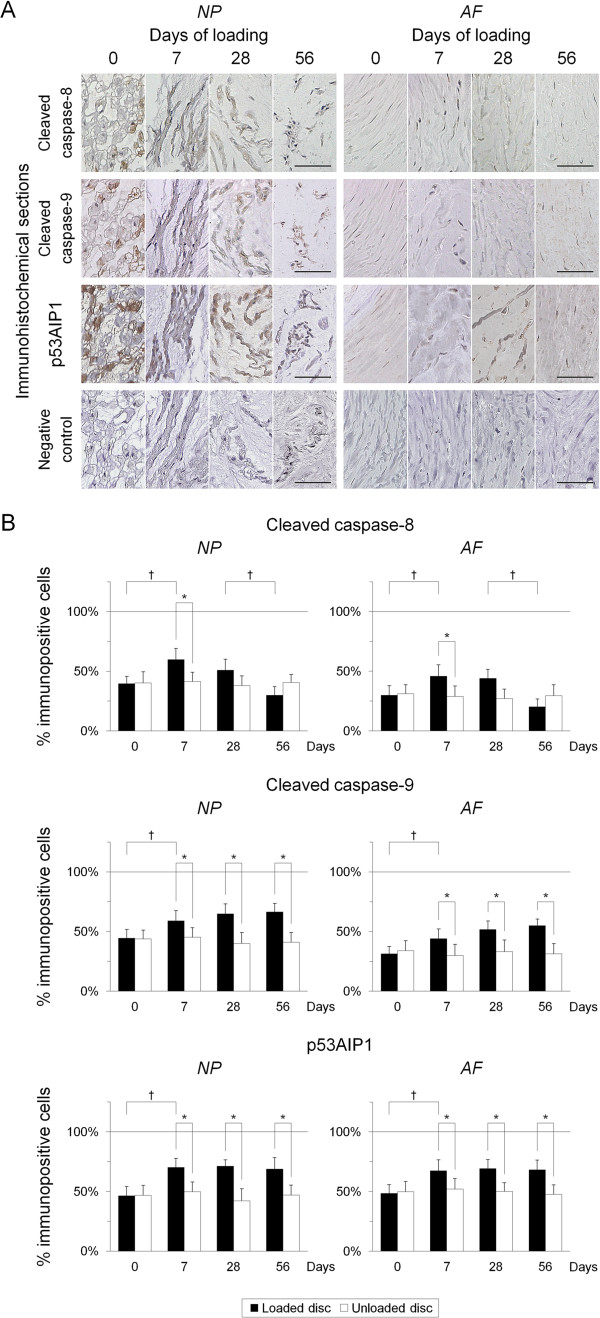
**Sustained static compression induces transient activation of apoptosis through the death receptor pathway and persistent activation of apoptosis through the p53-mediated mitochondrial pathway in intervertebral disc cells. (A)** Representative immunohistochemical sections for cleaved caspase-8, cleaved caspase-9, p53-regulated apoptosis-inducing protein 1 (p53AIP1), and negative control in the loaded disc nucleus pulposus (NP) and annulus fibrosus (AF) at 0, 7, 28, and 56 days of loading (bars, 50 μm). **(B)** Changes in the percentage of cleaved caspase-8-positive cells, cleaved caspase-9-positive cells, and p53AIP1-positive cells in the NP and AF at 0, 7, 28, and 56 days of loading. Data are mean ± standard deviation (*n* = 6). Two-way analysis of variance with the Tukey-Kramer *post hoc* test was used. *P < 0.05 when compared between loaded and unloaded conditions. ^†^*P* < 0.05 when compared between different time points.

To confirm whether static compression induces apoptosis through the death receptor pathway and/or the mitochondrial pathway, we performed immunohistochemistry for cleaved caspase-8 and cleaved caspase-9. Immunoreactivity for cleaved caspase-8 and cleaved caspase-9 was predominantly localized in the cytoplasm and stronger in the NP than in the AF (Figure 5A). The percentage of cells immunopositive for cleaved caspase-8 and cleaved caspase-9 was both higher at day 0 in the NP than in the AF. Interestingly, immunopositivity for cleaved caspase-8 and cleaved caspase-9 was higher than that for TUNEL. The percentage of cleaved capsase-8-positive cells increased only at day 7 (*P* < 0.05), whereas that of cleaved caspase-9-positive cells increased from day 7 through day 56 in the NP and AF (*P* < 0.05) (Figure 5B).

To clarify further whether mitochondrial apoptosis is mediated by p53, we performed immunohistochemistry for p53AIP1. Immunoreactivity for p53AIP1 was localized in the cytoplasm and greater in the NP than in the AF (Figure 5A). The percentage of cells immunopositive for p53AIP1 was comparable at day 0 in the NP and AF, and it was also higher than that for TUNEL. The percentage of p53AIP1-positive cells significantly increased from day 7 through day 56 in the NP and AF (*P* < 0.05) (Figure 5B).

### Sustained static compression induces consistently decreased expression of antiapoptotic proteins in intervertebral disc cells

**Figure 6:**
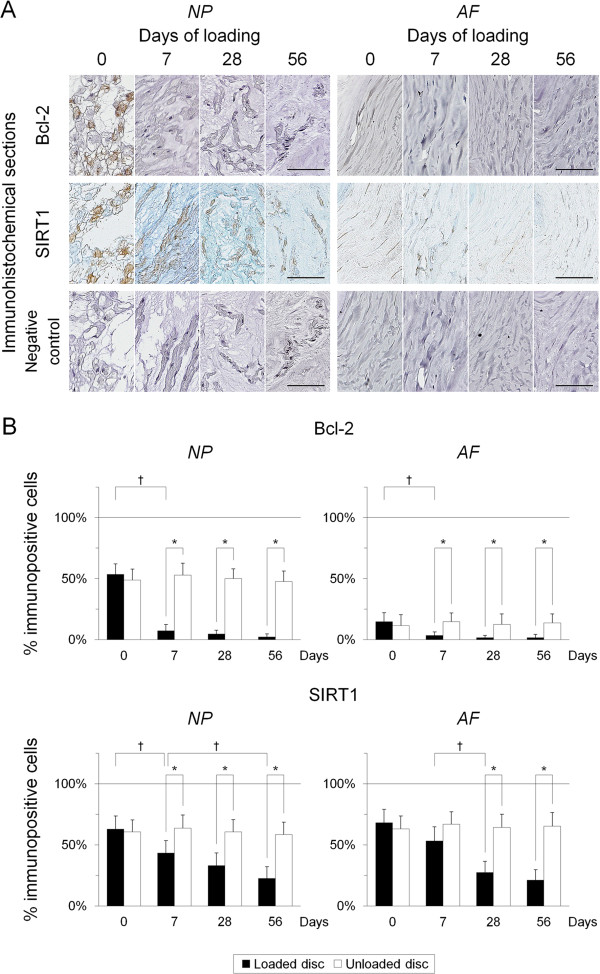
**Sustained static compression induces consistently decreased expression of antiapoptotic proteins in intervertebral disc cells. (A)** Representative immunohistochemical sections for B-cell lymphoma 2 (Bcl-2), silent mating-type information regulation 2 homolog 1 (SIRT1), and negative control in the loaded disc nucleus pulposus (NP) and annulus fibrosus (AF) at 0, 7, 28, and 56 days of loading (bars, 50 μm). **(B)** Changes in the percentage of Bcl-2-positive cells and SIRT1-positive cells in the NP and AF at 0, 7, 28, and 56 days of loading. Data are mean ± standard deviation (*n* = 6). Two-way analysis of variance with the Tukey-Kramer *post hoc* test was used. *P < 0.05 when compared between loaded and unloaded conditions. ^†^*P* < 0.05 when compared between different time points.

Finally, to seek for potential therapeutic targets for apoptosis in disc degeneration, we performed immunohistochemistry for antiapoptotic Bcl-2 and SIRT1. Immunoreactivity for Bcl-2 was localized in the cytoplasm, and that for SIRT1 was localized in the nucleus; both were stronger in the NP than in the AF (Figure 6A). The percentage of cells immunopositive for Bcl-2 was higher at day 0 in the NP than in the AF, whereas that for SIRT1 was constant in the NP and AF. The percentage of Bcl-2-positive cells decreased to nearly undetectable levels at day 7 and later time points in the NP and AF (*P* < 0.05). The percentage of SIRT1-positive cells progressively decreased from day 7 in the NP and from day 28 in the AF (*P* < 0.05) (Figure 6B).

## Discussion

Mechanical stress is one of the key contributors to intervertebral disc degeneration [6]. In the rodent tail model by Lotz and colleagues [23,24], static compression initiates apoptotic cell death in the inner AF, cartilage endplates, and then NP, which is worsened by increases in the magnitude (up to 1.3 MPa) and duration (up to 7 days) of loading. Furthermore, in the inner AF, static compression at 1.3 MPa for 24 hours induces increased reactivity for TUNEL and cytochrome *c* but not for FasL, indicating apoptosis induction primarily through the mitochondrial pathway [27]. However, these are the short-term effects. Few studies have explored the long-term changes in cell population and phenotype. Our rat tail model of disc degeneration induced by sustained static compression at 1.3 MPa for up to 56 days reproduces progressive radiographic disc height loss (>70%), lower T2-weighted magnetic resonance imaging (MRI) disc intensity, histologic degeneration [19], and extracellular matrix degradation with modified expression of catabolic enzymes and anticatabolic proteins [20]. In the current study, by using this model, we investigated the long-term progression of notochordal cell disappearance and apoptotic cell death in the disc. Furthermore, we studied time-dependent apoptotic signaling through the death receptor pathway and the mitochondrial pathway. This study elucidates the likely mechanisms behind decreased cellularity in static compression-induced disc degeneration.

Histomorphologic and immunofluorescent analysis demonstrated decreased disc NP and AF cells with compression. Eventually at day 56, only <50% of cells remained both in the NP and AF. Particularly, the decrease was notable at day 7 in NP cells with a notochordal phenotype—cytokeratin-8^+^ galectin-3^+^. This finding is consistent with the rabbit lumbar compression model study by Guehring and colleagues [31], showing more rapid reduction of cytokeratin-8^+^ cells than total NP cells by mechanical compression. Their study suggested increased sclerosis and fibrosis in the endplates, leading to the loss of nutrient supply, which could explain why notochordal cells decrease in number and lose their phenotype in compressive stress-induced disc degeneration. In addition, an MRI study using a contrast agent implied impairment of diffusion of nutrients from the periphery through the endplates with sustained mechanical loading [32]. Therefore, the static compression model may be associated with nutrient deprivation. Notochordal cells require a higher amount of energy to survive and are more vulnerable to nutrient deprivation than do non-notochordal, chondrocyte-like cells [33]. Thus, notochordal cells appear to be less resistant to mechanical loading and related nutrient deprivation than do non-notochordal cells. However, how these stresses affect notochordal cells and reduce their numbers was largely unknown. Hence, we next examined the apoptosis of notochordal cells.

TUNEL staining and immunohistochemistry for cleaved caspase-3 demonstrated increased involvement of apoptosis with compression, which concurs with human [18,34] and other static compression studies [23-27]. Then, immunohistochemistry for cleaved caspase-8 and cleaved caspase-9 showed transient activation of death receptor signaling and persistent activation of mitochondrial signaling in static compression-induced apoptosis. Caspase-dependent apoptosis requires proteolytic cleavage; therefore, the presence of cleaved caspases indicates activated apoptosis through their own pathways [12]. In the context of the mouse model study by Rannou and colleagues [27] describing increased mitochondrial cytochrome *c* release but not death ligand, FasL, expression in the AF under 24-hour static compression, our longitudinal, longer-term observation of caspase cleavage products provides more direct evidence regarding the involved apoptotic pathways during disc degeneration. Furthermore, immunohistochemistry for p53AIP1 exhibited that the observed mitochondrial apoptosis was consistently mediated by p53 signaling. p53AIP1 expression itself indicates induction of p53-dependent mitochondrial apoptosis [16,17]. Although few studies have reported p53AIP1 induction in the disc, a human chondrocyte study showed highly expressed p53AIP1 in osteoarthritic cartilage and increased p53AIP1 expression by shear strain [35]. Taken together, the present results indicate that apoptosis is involved in static compression-induced disc degeneration, which is induced by a combination of signals through the death receptor pathway and more dominantly through the p53-mediated mitochondrial pathway.

Immunohistochemistry for Bcl-2 and SIRT1 demonstrated that expression of these antiapoptotic proteins decreased with compression, which also concurs with reported evidence [36,37]. Overexpression of Bcl-2 limits disc cell apoptosis and messenger RNA downregulation of *aggrecan* and *collagen type 2* under serum starvation [38]. SIRT1 activation by resveratrol inhibits nitric oxide-induced mitochondrial apoptosis in chondrocytes, showing decreased Bax and increased Bcl-2 [37]. Overexpression of SIRT1 suppresses catabolic gene upregulation induced by interleukin-1β [39]. Thus, the consistently decreased Bcl-2 and SIRT1 expression may contribute to the acceleration of apoptotic cell death in static compression-induced disc degeneration.

**Figure 7:**
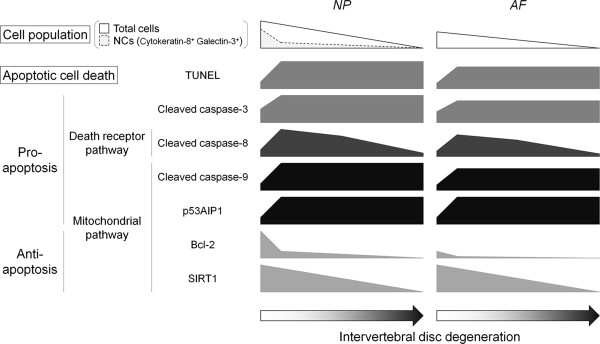
**Schematic illustration summarizing static compression-induced notochordal cell disappearance and apoptotic cell death in the intervertebral disc.** The number of disc nucleus pulposus (NP) and annulus fibrosus (AF) cells decreases with compression; particularly, the decrease is notable in larger, vacuolated cells with a notochordal phenotype, cytokeratin-8^+^ galectin-3^+^. Subsequently, the proportion of apoptotic terminal deoxynucleotidyl transferase dUTP nick-end labeling (TUNEL)-positive cells and cleaved caspase-3-positive cells increases throughout. This apoptosis induction is mediated transiently through the death receptor pathway, as determined by immunopositivity for cleaved caspase-8, and persistently through the p53-dependent mitochondrial pathway, as identified by immunopositivity for cleaved caspase-9 and p53-regulated apoptosis-inducing protein 1 (p53AIP1). The percentage of cells expressing antiapoptotic proteins, B-cell lymphoma 2 (Bcl-2), and silent mating-type information regulation 2 homolog 1 (SIRT1), decreases consistently. The increased proapoptotic and decreased antiapoptotic proteins are an indication of static compression-induced disc cell death and degeneration.

The cell decrease in this rat tail model is summarized in Figure 7. Although the majority of cells with a notochordal phenotype disappeared at day 7, the proportion of apoptotic cells without notochordal phenotype substantially increased. This raises two questions. The first question is how notochordal cells are actually lost—cell death or phenotypic loss? A tamoxifen-inducible *ShhcreER*^*T2*^ mouse study showed that the entire NP cell population, even in the adult, descends from the notochord [7]. In a mouse disc injury model, annular puncture induces transformation of the notochordal NP into a chondrogenic and subsequently fibrocartilaginous phenotype [40]. Our cell count data, which showed a transient increase in non-notochordal NP cells, may also indicate a phenotypic change with differentiation of notochordal into non-notochordal cells by mechanical stress.

Somewhat inconsistent TUNEL and immunohistochemical staining results were seen in the NP at day 0. Although TUNEL-positivity was lower, immunoreactivity for apoptotic and antiapoptotic proteins was stronger and immunopositivity for cleaved caspases and Bcl-2 was higher. The higher positivity for cleaved caspases versus TUNEL is similar to the higher positivity for cytochrome *c* versus TUNEL in the mouse-model study by Rannou and colleagues [27]. A previous comparative study of apoptosis detection methods concluded that immunohistochemical staining for cleaved caspase-3 is a more sensitive and reliable method for detecting and quantifying apoptosis than is TUNEL staining [41]. That study showed that positivity for cleaved caspase-3 was approximately threefold higher than that for TUNEL staining but was close to that for morphologically identified apoptotic cells. Therefore, our findings potentially indicate high baseline levels of apoptotic signals in notochordal cells, suggesting a preapoptotic state. Our 56-day observation did not detect additional increase in apoptosis of notochordal cells in unloaded control discs. Longer-term studies may be required to investigate age-related increases in the apoptosis, as notochordal cell disappearance was reported previously in a >2-year rabbit study [42]. In addition, the decrease in Bcl-2 expression was more pronounced between days 0 and 7, suggesting an important role of Bcl-2 in notochordal cell homeostasis. Thus, our findings lend support to the view that notochordal cell disappearance is associated with apoptosis. However, no direct evidence exists regarding whether the possible phenotypic transition from notochordal to non-notochordal is associated with apoptotic signaling. Further studies are needed to understand the mechanism of notochordal cell disappearance.

The second question concerns with the causative roles of notochordal cell disappearance and increased apoptosis of non-notochordal cells in intervertebral disc degeneration. The observed finding at day 7 raises the possibility that cell death during disc degeneration is driven by non-notochordal cells, whereas the loss of notochordal cells is coincidental. Discs of some species (for example, bovine, ovine, and equine) lose notochordal cells before or rapidly after birth; however, their discs show few signs of degenerative changes, even in adulthood [6]. This indicates that the loss of notochordal cells is not always an indication of disc degeneration. It is still controversial whether the loss of notochordal cells is a part of species-specific development or of the degenerative process in the human disc. *In vitro*, notochordal cell-conditioned medium protects non-notochordal cells from FasL-mediated apoptosis and interleukin-1β-mediated inflammation [43]. In addition, notochordal cells produce a larger amount of proteoglycans than do non-notochordal cells [44] and stimulate non-notochordal cells to produce proteoglycans [45]. Taken together with the susceptibility of notochordal cells by mechanical [31] and nutritional [33] stress, notochordal cells potentially lead to altering their phenotype and/or cell death under static compression, whereas non-notochordal cells may be actively involved in subsequent disc degeneration. This rat tail static compression model mimics notochordal cell disappearance and apoptotic cell death in human intervertebral disc aging and degeneration.

A major limitation of the study is that the static-compression model simulates nonphysiological conditions: immobility, extended pressure, and absence of trauma [19,20,30]. Lower levels of inflammation in this model, as shown previously [20], may relate to less involvement of apoptosis through the death receptor pathway. Another limitation is that the results of the staining assays are not completely quantitative. Our analysis of apoptotic signaling was limited to immunohistochemistry because it was difficult to collect sufficient protein extracts for Western blotting from severely degenerated discs. In addition, this study does not probe all apoptotic pathways. For example, Bcl-2 family members can function to activate caspase-12, inducing endoplasmic reticulum-mediated apoptosis [12].

The specificity of cytokeratin-8 and galectin-3 as exclusive notochordal cell markers is not certain. In the current study, although 67.4% of cells co-immunopositive for cytokeratin-8 and galectin-3 with stronger immunoreactivity were observed in the NP at day 0, 34.0% of double-immunopositive cells were detected in the AF as well. This is consistent with Western blot results reported by Oguz and colleagues [46] showing that galectin-3 expression is highest in the rat NP, but AF and cartilage tissues also express galectin-3. In addition, microarray studies found that adult bovine and human NP tissues still express cytokeratin-8 [47,48]. Cells positive for cytokeratins and galectin-3 are present in a considerable fraction of adult human lumbar discs [49], raising the question whether notochordal cells are truly lost in the disc during postnatal life. Cytokeratin-8 and galectin-3 are useful markers; however, they are by no means exclusively specific for notochordal cells. The establishment of more specific notochordal cell markers is required as still no definitive markers of notochordal cells are known [50].

This study describes the predominance of the mitochondrial pathway of apoptosis over the death receptor pathway during disc degeneration. An *in vivo* study using a rabbit annular puncture model demonstrated that knockdown of caspase-3 by small interference RNA results in delayed disc degeneration [51]. However, caspase inhibition has shown induction of alternative cell death-related programs, including necrosis, autophagy, and senescence [52,53]. Taken together, overexpression of antiapoptotic proteins through the mitochondrial pathway (for example, Bcl-2) may represent a specific, effective molecular treatment option in degenerative disc disease. Future mechanistic studies must be conducted.

## Conclusions

This rat tail static compression model mimics notochordal cell disappearance and apoptotic cell death in human intervertebral disc aging and degeneration. In early degenerative stages, the number of disc cells, particularly cells with a notochordal phenotype, drastically decreases, whereas the proportion of apoptotic cells increases. In late stages, disc cell numbers further decline because of apoptosis. This apoptosis induction is mediated transiently through the death receptor pathway and persistently through the p53-dependent mitochondrial pathway. The increased proapoptotic and decreased antiapoptotic proteins observed at every time point are an indication of static compression-induced disc cell death and degeneration.

## Abbreviations

AF: Annulus fibrosus; Bad: Bcl-2-associated agonist of cell death; Bax: Bcl-2-associated X protein; Bcl-2: B-cell lymphoma 2; Bid: BH3 interacting domain death agonist; C: caudal; DAPI: 4′,6-diamidino-2-phenylindole; FasL: Fas ligand; Mdm2: murine double-minute 2; Mdm4: murine double-minute 4; MRI: magnetic resonance imaging; NP: nucleus pulposus; p53AIP1: p53-regulated apoptosis-inducing protein 1; SIRT1: silent mating type information regulation 2 homolog 1; TUNEL: terminal deoxynucleotidyl transferase dUTP nick-end labeling.

## Competing interests

The authors declare that they have no competing interests.

## Authors’ contributions

TY conceived of the study, secured funding, carried out the animal model studies, participated in its design and coordination and the interpretation of the data, performed the statistical analysis, and drafted the manuscript. HH carried out the animal model studies and participated in the interpretation of the data. KK, KM, TT, ZZ, KT, TM, RK, and MK participated in the interpretation of the data. KN secured funding, participated in the design and coordination of the study and the interpretation of the data, and helped to draft the manuscript. All authors read and approved the final manuscript.

## References

[B1] Strine TW Hootman JM US national prevalence and correlates of low back and neck pain among adults Arthritis Rheum 2007 16 656 665 10.1002/art.22684 17471542

[B2] Luo X Pietrobon R Sun SX Liu GG Hey L Estimates and patterns of direct health care expenditures among individuals with back pain in the United States Spine (Phila Pa 1976) 2004 16 79 86 10.1097/01.BRS.0000105527.13866.0F 14699281

[B3] Stewart WF Ricci JA Chee E Morganstein D Lipton R Lost productive time and cost due to common pain conditions in the US workforce JAMA 2003 16 2443 2454 10.1001/jama.290.18.2443 14612481

[B4] Livshits G Popham M Malkin I Sambrook PN Macgregor AJ Spector T Williams FM Lumbar disc degeneration and genetic factors are the main risk factors for low back pain in women: the UK Twin Spine Study Ann Rheum Dis 2011 16 1740 1745 10.1136/ard.2010.137836 21646416 PMC3171106

[B5] Hunter CJ Matyas JR Duncan NA The notochordal cell in the nucleus pulposus: a review in the context of tissue engineering Tissue Eng 2003 16 667 677 10.1089/107632703768247368 13678445

[B6] Alini M Eisenstein SM Ito K Little C Kettler AA Masuda K Melrose J Ralphs J Stokes I Wilke HJ Are animal models useful for studying human disc disorders/degeneration? Eur Spine J 2008 16 2 19 10.1007/s00586-007-0414-y 17632738 PMC2365516

[B7] Choi KS Cohn MJ Harfe BD Identification of nucleus pulposus precursor cells and notochordal remnants in the mouse: implications for disk degeneration and chordoma formation Dev Dyn 2008 16 3953 3958 10.1002/dvdy.21805 19035356 PMC2646501

[B8] Urban JP Roberts S Degeneration of the intervertebral disc Arthritis Res Ther 2003 16 120 130 12723977 10.1186/ar629 PMC165040

[B9] Antoniou J Steffen T Nelson F Winterbottom N Hollander AP Poole RA Aebi M Alini M The human lumbar intervertebral disc: evidence for changes in the biosynthesis and denaturation of the extracellular matrix with growth, maturation, ageing, and degeneration J Clin Invest 1996 16 996 1003 10.1172/JCI118884 8770872 PMC507515

[B10] Boos N Weissbach S Rohrbach H Weiler C Spratt KF Nerlich AG Classification of age-related changes in lumbar intervertebral discs: 2002 Volvo Award in basic science Spine (Phila Pa 1976) 2002 16 2631 2644 10.1097/00007632-200212010-00002 12461389

[B11] Fuchs Y Steller H Programmed cell death in animal development and disease Cell 2011 16 742 758 10.1016/j.cell.2011.10.033 22078876 PMC4511103

[B12] Jin Z El-Deiry WS Overview of cell death signaling pathways Cancer Biol Ther 2005 16 139 163 10.4161/cbt.4.2.1508 15725726

[B13] Luo J Nikolaev AY Imai S Chen D Su F Shiloh A Guarente L Gu W Negative control of p53 by Sir2alpha promotes cell survival under stress Cell 2001 16 137 148 10.1016/S0092-8674(01)00524-4 11672522

[B14] Vaziri H Dessain SK Ng Eaton E Imai SI Frye RA Pandita TK Guarente L Weinberg RA hSIR2(SIRT1) functions as an NAD-dependent p53 deacetylase Cell 2001 16 149 159 10.1016/S0092-8674(01)00527-X 11672523

[B15] Toledo F Krummel KA Lee CJ Liu CW Rodewald LW Tang M Wahl GM A mouse p53 mutant lacking the proline-rich domain rescues Mdm4 deficiency and provides insight into the Mdm2-Mdm4-p53 regulatory network Cancer Cell 2006 16 273 285 10.1016/j.ccr.2006.03.014 16616333

[B16] Oda K Arakawa H Tanaka T Matsuda K Tanikawa C Mori T Nishimori H Tamai K Tokino T Nakamura Y Taya Y p53AIP1, a potential mediator of p53-dependent apoptosis, and its regulation by Ser-46-phosphorylated p53 Cell 2000 16 849 862 10.1016/S0092-8674(00)00073-8 11030628

[B17] Matsuda K Yoshida K Taya Y Nakamura K Nakamura Y Arakawa H p53AIP1 regulates the mitochondrial apoptotic pathway Cancer Res 2002 16 2883 2889 12019168

[B18] Gruber HE Hanley EN Jr Analysis of aging and degeneration of the human intervertebral disc. Comparison of surgical specimens with normal controls Spine (Phila Pa 1976) 1998 16 751 757 10.1097/00007632-199804010-00001 9563104

[B19] Yurube T Nishida K Suzuki T Kaneyama S Zhang Z Kakutani K Maeno K Takada T Fujii M Kurosaka M Doita M Matrix metalloproteinase (MMP)-3 gene up-regulation in a rat tail compression loading-induced disc degeneration model J Orthop Res 2010 16 1026 1032 20162718 10.1002/jor.21116

[B20] Yurube T Takada T Suzuki T Kakutani K Maeno K Doita M Kurosaka M Nishida K Rat tail static compression model mimics extracellular matrix metabolic imbalances of matrix metalloproteinases, aggrecanases, and tissue inhibitors of metalloproteinases in intervertebral disc degeneration Arthritis Res Ther 2012 16 R51 10.1186/ar3764 22394620 PMC3446417

[B21] Sztrolovics R Alini M Roughley PJ Mort JS Aggrecan degradation in human intervertebral disc and articular cartilage Biochem J 1997 16 235 241 9337874 10.1042/bj3260235 PMC1218660

[B22] Roberts S Caterson B Menage J Evans EH Jaffray DC Eisenstein SM Matrix metalloproteinases and aggrecanase: their role in disorders of the human intervertebral disc Spine (Phila Pa 1976) 2000 16 3005 3013 10.1097/00007632-200012010-00007 11145811

[B23] Lotz JC Colliou OK Chin JR Duncan NA Liebenberg E Compression-induced degeneration of the intervertebral disc: an in vivo mouse model and finite-element study Spine (Phila Pa 1976) 1998 16 2493 2506 10.1097/00007632-199812010-00004 9854748

[B24] Lotz JC Chin JR Intervertebral disc cell death is dependent on the magnitude and duration of spinal loading Spine (Phila Pa 1976) 2000 16 477 1483 10.1097/00007632-200006150-00005 10851095

[B25] Hutton WC Ganey TM Elmer WA Kozlowska E Ugbo JL Doh ES Whitesides TE Jr Does long-term compressive loading on the intervertebral disc cause degeneration? Spine (Phila Pa 1976) 2000 16 2993 3004 10.1097/00007632-200012010-00006 11145810

[B26] Kroeber MW Unglaub F Wang H Schmid C Thomsen M Nerlich A Richter W New in vivo animal model to create intervertebral disc degeneration and to investigate the effects of therapeutic strategies to stimulate disc regeneration Spine (Phila Pa 1976) 2002 16 2684 2690 10.1097/00007632-200212010-00007 12461394

[B27] Rannou F Lee TS Zhou RH Chin J Lotz JC Mayoux-Benhamou MA Barbet JP Chevrot A Shyy JY Intervertebral disc degeneration: the role of the mitochondrial pathway in annulus fibrosus cell apoptosis induced by overload Am J Pathol 2004 16 915 924 10.1016/S0002-9440(10)63179-3 14982845 PMC1613264

[B28] Hughes PC Tanner JM The assessment of skeletal maturity in the growing rat J Anat 1970 16 371 402 4315144 PMC1233709

[B29] Iatridis JC Mente PL Stokes IA Aronsson DD Alini M Compression-induced changes in intervertebral disc properties in a rat tail model Spine (Phila Pa 1976) 1999 16 996 1002 10.1097/00007632-199905150-00013 10332792

[B30] Yurube T Takada T Hirata H Kakutani K Maeno K Zhang Z Yamamoto J Doita M Kurosaka M Nishida K Modified house-keeping gene expression in a rat tail compression loading-induced disc degeneration model J Orthop Res 2011 16 1284 1290 10.1002/jor.21406 21387398

[B31] Guehring T Nerlich A Kroeber M Richter W Omlor GW Sensitivity of notochordal disc cells to mechanical loading: an experimental animal study Eur Spine J 2010 16 113 121 10.1007/s00586-009-1217-0 19936803 PMC2899741

[B32] Arun R Freeman BJ Scammell BE McNally DS Cox E Gowland P ISSLS Prize Winner: What influence does sustained mechanical load have on diffusion in the human intervertebral disc?: an in vivo study using serial postcontrast magnetic resonance imaging Spine (Phila Pa 1976) 2009 16 2324 2337 10.1097/BRS.0b013e3181b4df92 19755934

[B33] Guehring T Wilde G Sumner M Grunhagen T Karney GB Tirlapur UK Urban JP Notochordal intervertebral disc cells: sensitivity to nutrient deprivation Arthritis Rheum 2009 16 1026 1034 10.1002/art.24407 19333932

[B34] Trout JJ Buckwalter JA Moore KC Ultrastructure of the human intervertebral disc: II. Cells of the nucleus pulposus Anat Rec 1982 16 307 314 10.1002/ar.1092040403 7181135

[B35] Hashimoto S Nishiyama T Hayashi S Fujishiro T Takebe K Kanzaki N Kuroda R Kurosaka M Role of p53 in human chondrocyte apoptosis in response to shear strain Arthritis Rheum 2009 16 2340 2349 10.1002/art.24706 19644890

[B36] Kim HA Lee YJ Seong SC Choe KW Song YW Apoptotic chondrocyte death in human osteoarthritis J Rheumatol 2000 16 455 462 10685814

[B37] Takayama K Ishida K Matsushita T Fujita N Hayashi S Sasaki K Tei K Kubo S Matsumoto T Fujioka H Kurosaka M Kuroda R SIRT1 regulation of apoptosis of human chondrocytes Arthritis Rheum 2009 16 2731 2740 10.1002/art.24864 19714620

[B38] Sudo H Minami A Regulation of apoptosis in nucleus pulposus cells by optimized exogenous Bcl-2 overexpression J Orthop Res 2010 16 1608 1613 10.1002/jor.21185 20589931

[B39] Matsushita T Sasaki H Takayama K Ishida K Matsumoto T Kubo S Matsuzaki T Nishida K Kurosaka M Kuroda R The overexpression of SIRT1 inhibited osteoarthritic gene expression changes induced by interleukin-1beta in human chondrocytes J Orthop Res 2013 16 531 537 10.1002/jor.22268 23143889

[B40] Yang F Leung VY Luk KD Chan D Cheung KM Injury-induced sequential transformation of notochordal nucleus pulposus to chondrogenic and fibrocartilaginous phenotype in the mouse J Pathol 2009 16 113 121 10.1002/path.2519 19288580

[B41] Duan WR Garner DS Williams SD Funckes-Shippy CL Spath IS Blomme EA Comparison of immunohistochemistry for activated caspase-3 and cleaved cytokeratin 18 with the TUNEL method for quantification of apoptosis in histological sections of PC-3 subcutaneous xenografts J Pathol 2003 16 221 228 10.1002/path.1289 12533835

[B42] Sowa G Vadala G Studer R Kompel J Iucu C Georgescu H Gilbertson L Kang J Characterization of intervertebral disc aging: longitudinal analysis of a rabbit model by magnetic resonance imaging, histology, and gene expression Spine (Phila Pa 1976) 2008 16 1821 1828 10.1097/BRS.0b013e31817e2ce3 18670334

[B43] Erwin WM Islam D Inman RD Fehlings MG Tsui FW Notochordal cells protect nucleus pulposus cells from degradation and apoptosis: implications for the mechanisms of intervertebral disc degeneration Arthritis Res Ther 2011 16 R215 10.1186/ar3548 22206702 PMC3334668

[B44] Cappello R Bird JL Pfeiffer D Bayliss MT Dudhia J Notochordal cell produce and assemble extracellular matrix in a distinct manner, which may be responsible for the maintenance of healthy nucleus pulposus Spine (Phila Pa 1976) 2006 16 873 882 discussion 883 10.1097/01.brs.0000209302.00820.fd 16622374

[B45] Erwin WM Inman RD Notochord cells regulate intervertebral disc chondrocyte proteoglycan production and cell proliferation Spine (Phila Pa 1976) 2006 16 1094 1099 10.1097/01.brs.0000216593.97157.dd 16648742

[B46] Oguz E Tsai TT Di Martino A Guttapalli A Albert TJ Shapiro IM Risbud MV Galectin-3 expression in the intervertebral disc: a useful marker of the notochord phenotype? Spine (Phila Pa 1976) 2007 16 9 16 10.1097/01.brs.0000250302.74574.98 17202886

[B47] Minogue BM Richardson SM Zeef LA Freemont AJ Hoyland JA Transcriptional profiling of bovine intervertebral disc cells: implications for identification of normal and degenerate human intervertebral disc cell phenotypes Arthritis Res Ther 2010 16 R22 10.1186/ar2929 20149220 PMC2875656

[B48] Gilson A Dreger M Urban JP Differential expression level of cytokeratin 8 in cells of the bovine nucleus pulposus complicates the search for specific intervertebral disc cell markers Arthritis Res Ther 2010 16 R24 10.1186/ar2931 20152014 PMC2875658

[B49] Weiler C Nerlich AG Schaaf R Bachmeier BE Wuertz K Boos N Immunohistochemical identification of notochordal markers in cells in the aging human lumbar intervertebral disc Eur Spine J 2010 16 1761 1770 10.1007/s00586-010-1392-z 20372940 PMC2989227

[B50] Risbud MV Shapiro IM Notochordal cells in the adult intervertebral disc: new perspective on an old question Crit Rev Eukaryot Gene Expr 2011 16 29 41 10.1615/CritRevEukarGeneExpr.v21.i1.30 21967331 PMC3187872

[B51] Sudo H Minami A Caspase 3 as a therapeutic target for regulation of intervertebral disc degeneration in rabbits Arthritis Rheum 2011 16 1648 1657 10.1002/art.30251 21305515

[B52] Rebbaa A Zheng X Chou PM Mirkin BL Caspase inhibition switches doxorubicin-induced apoptosis to senescence Oncogene 2003 16 2805 2811 10.1038/sj.onc.1206366 12743603

[B53] Vandenabeele P Vanden Berghe T Festjens N Caspase inhibitors promote alternative cell death pathways Sci STKE 2006 16 pe44 17062895 10.1126/stke.3582006pe44

